# Cross clade reactive plasma anti-V3 antibodies in human immunodeficiency virus type-1 infected individuals develop with time

**DOI:** 10.1186/1471-2334-12-S1-P24

**Published:** 2012-05-04

**Authors:** Raiees Andrabi, Manju Bala, Kalpana Luthra

**Affiliations:** 1 Department of Biochemistry, All India Institute of Medical Sciences, New Delhi, India; 2 Regional STD Teaching Training & Research Centre, Safdarjang Hospital, New Delhi, India

## Background

Subtype-C alone accounts for approximately 50% of global and more than 95% human immunodeficiency virus-1 (HIV-1) infection in India. Identification of antigenic epitopes that induce antibodies with cross-clade activity will be crucial to address the HIV-1 viral diversity.

## Methods

80 HIV-1 infected drug naive patients were recruited for this study. The study was approved by the institute ethics committee and informed consent was obtained from all the participants. The relative binding of anti-V3 polyclonal plasma antibodies to 35 mer consensus-B and C V3 peptides was done by ELISA binding assay. Statistical analysis was performed by Graphpad Prism 5.

## Results

Assessment of the relative binding revealed that 86% (69/80) and 99% (79/80) of the plasma were able to reach an IC50 binding titer with V3B peptide and V3C peptides respectively; with substantially low antibody titers that bind to V3B than V3C (mean IC50, V3B=2736 versus V3C=12612) (p<0.0001). The finding suggests that although majority of the antibodies were subtype specific, a good proportion of cross reactive anti-V3 antibodies also exist in these plasma (mean=23% range=0.11-97%). We observed a positive correlation between percent cross reactive anti-V3 antibodies and days from first diagnosis (p=0.008) while no such association was found with other clinical and immunological parameters like plasma viral load (p=0.24), CD4 count (p=0.34) and total plasma IgG levels (p=0.45).

## Conclusions

This is the first study to demonstrate the presence of cross-clade reactive anti-V3 antibodies and their association with time in the plasma of HIV-1 infected Asian Indians from north India.

